# Supplementary material for: Improved Peptide Backbone Fragmentation Is the Primary Advantage of MS-Cleavable Crosslinkers
Source: Anal Chem. 2022 May 25;94(22):7779–86. doi: 10.1021/acs.analchem.1c05266 (PMC9178559; doi:10.1021/acs.analchem.1c05266)
Supplement: Supplementary file 1 — ac1c05266_si_001.pdf [file ac1c05266_si_001.pdf]

Supplementary Materials for

Improved peptide backbone fragmentation is the primary advantage of MS-cleavable crosslinkers

Lars Kolbowski[*,1], Swantje Lenz[*,1], Lutz Fischer[1], Ludwig R Sinn[1], Francis J O'Reilly[1], Juri Rappsilber[†,1,2]

[*] These authors contributed equally to this work
[1] Technische Universität Berlin, Chair of Bioanalytics, 10623 Berlin, Germany
[2] University of Edinburgh, Wellcome Centre for Cell Biology, Edinburgh EH9 3BF, UK
[†] Corresponding author email address: juri.rappsilber@tu-berlin.de

This file includes:

Supplementary Figures S1-11

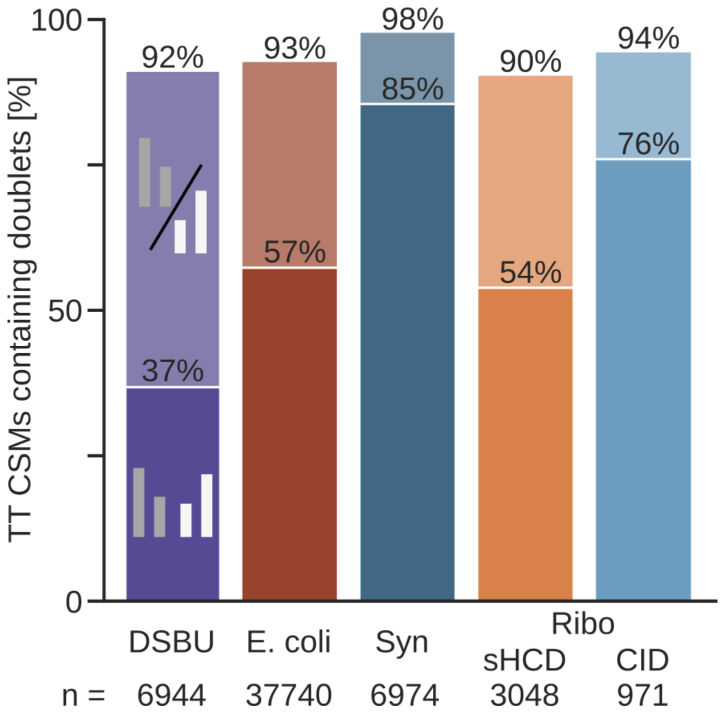

Figure S1. Ratio of identified target-target (TT) CSMs (self and heteromeric) that contain one (lighter color) or both (darker color) peptide doublets in each dataset (5% CSM-level FDR). DSSO datasets using sHCD are shown in orange-red while CID-MS3 based methods are in blue colors. The DSBU data (purple colors) is also acquired using sHCD.

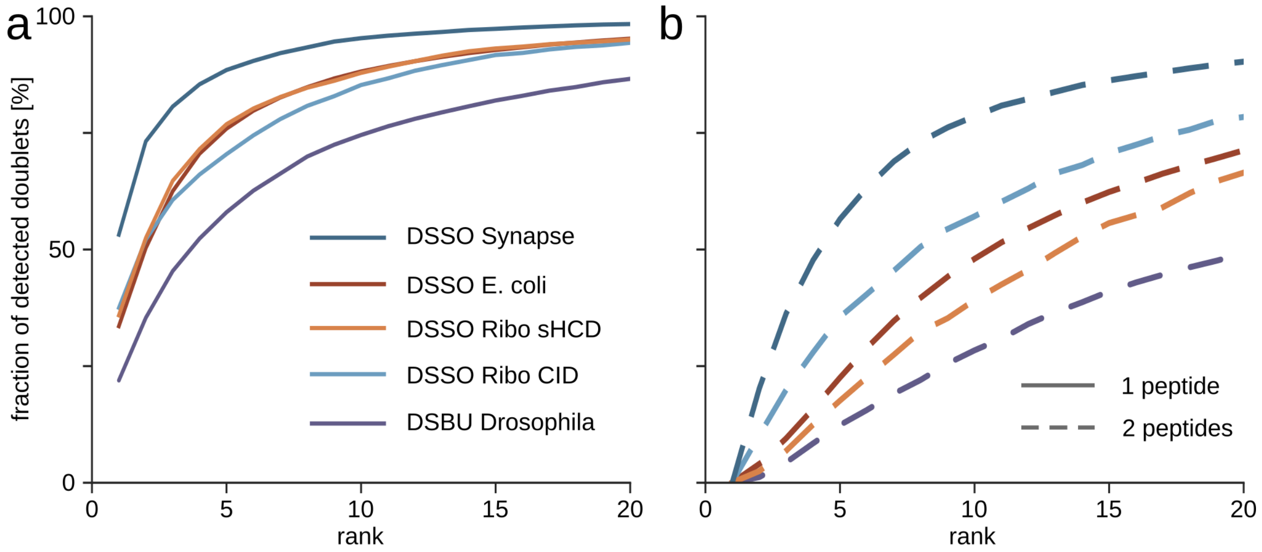

Figure S2. Fraction of detected doublets from S1 passing each intensity rank cut-off for the one (b) and both (c) peptides. Shown is the cumulative proportion of CSMs containing doublets.

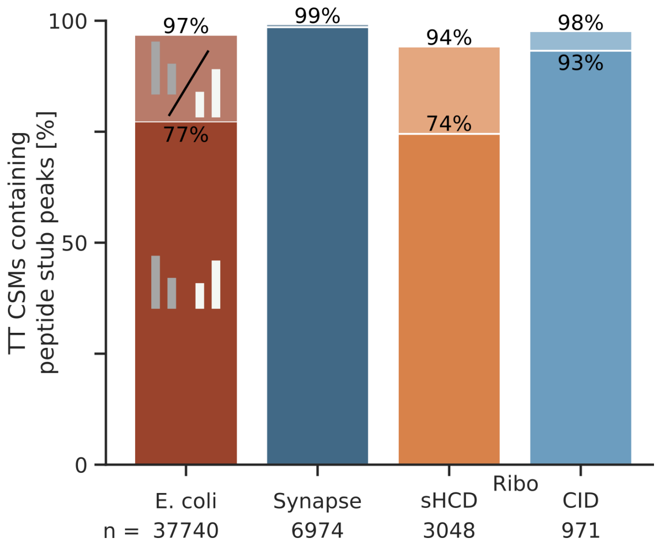

Figure S3. Ratio of identified target-target (TT) CSMs that contain at least one peptide stub peak for one (lighter color) or both (darker color) crosslinked peptides across datasets (5% CSM-level FDR).

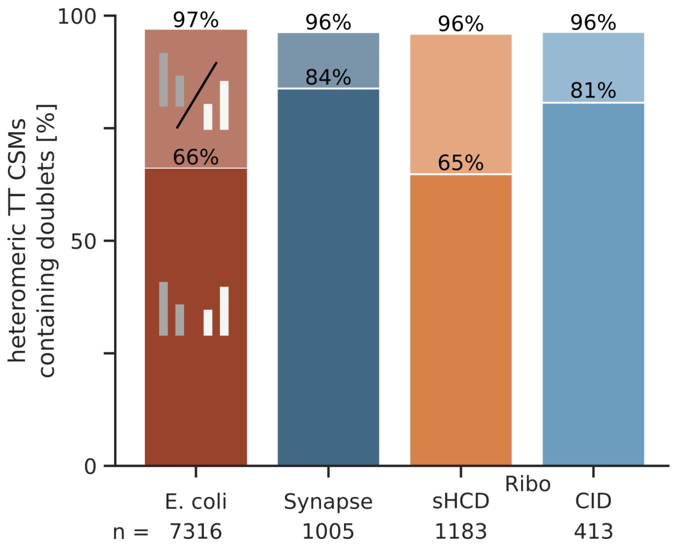

Figures S4. Ratio of identified heteromeric target-target (TT) CSMs that contain one (lighter color) or both (darker color) peptide doublets across datasets (5% CSM-level FDR).

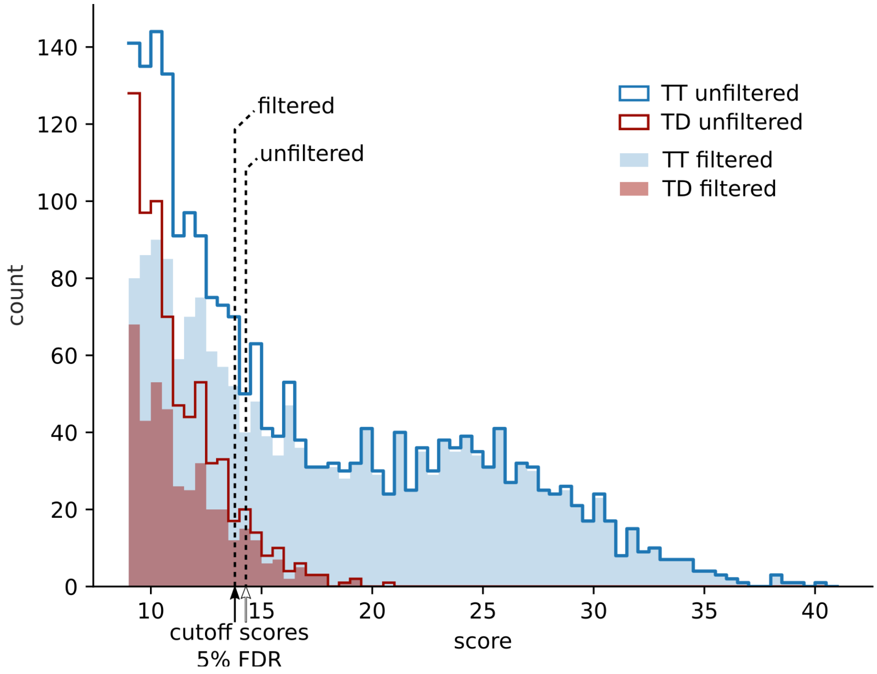

Figure S5. Score distribution of heteromeric matches in the Ribosome HCD dataset. Shown is the distribution of targets and target-decoy matches with and without filtering for peptide doublets. Arrows show the resulting score cutoffs at 5% FDR.

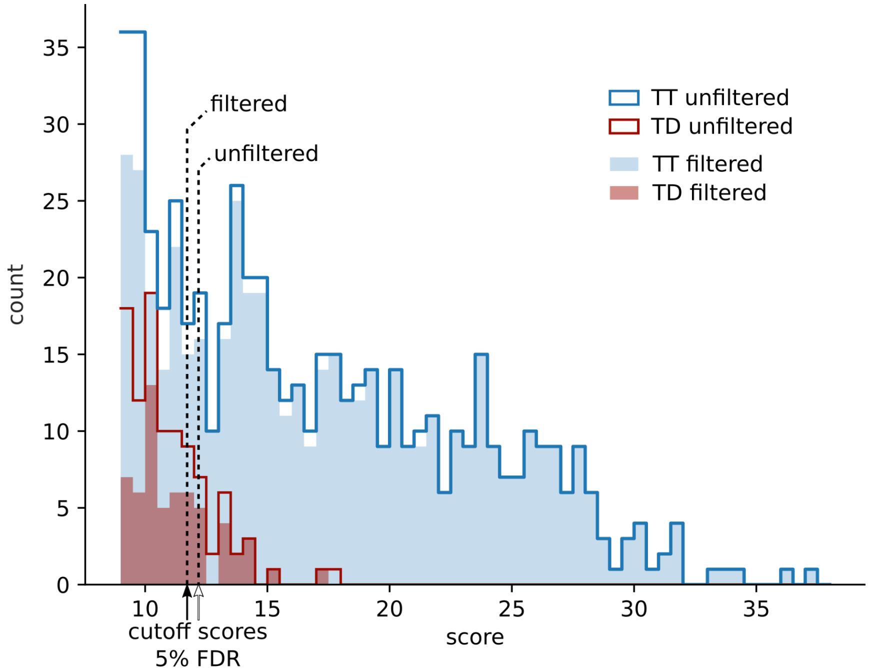

Figure S6. Score distribution of heteromeric matches in the Ribosome CID dataset. Shown is the distribution of targets and target-decoy matches with and without filtering for peptide doublets. Arrows show the resulting score cutoffs at 5% FDR.

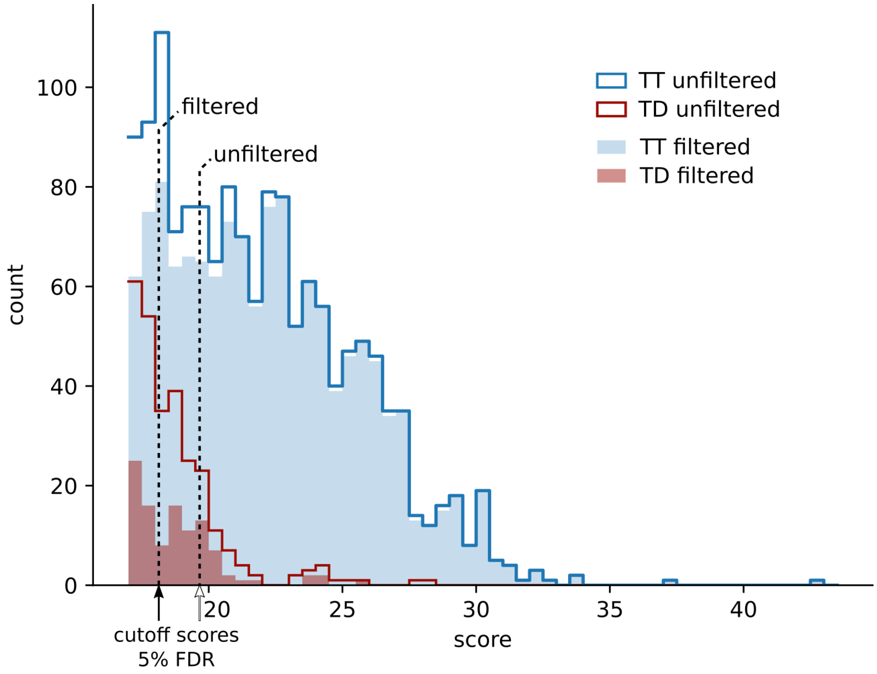

Figure S7. Score distribution of heteromeric matches in the Synapse dataset. Shown is the distribution of targets and target-decoy matches with and without filtering for peptide doublets. Arrows show the resulting score cutoffs at 5% FDR.

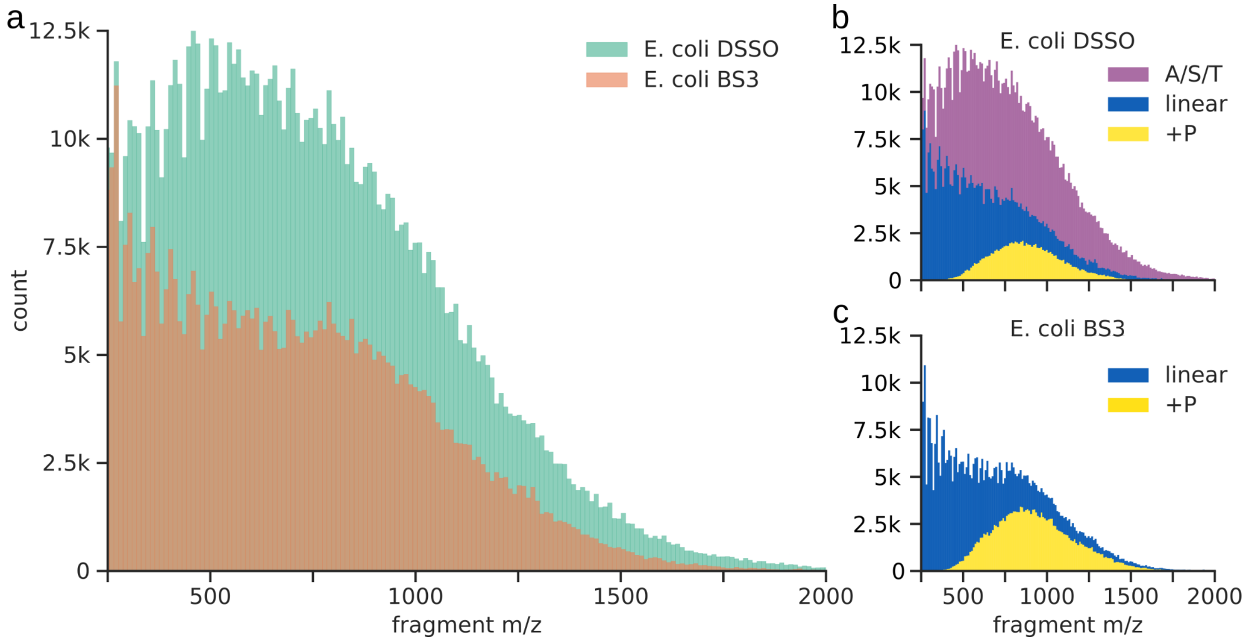

Figure S8. (a) Histogram of sequence fragments present in common CSMs (n=12919) between the *E. coli* DSSO and the BS3 dataset. (b) and (c) show the distributions from (a) split into the linear, the full second peptide containing (+P) and for the DSSO dataset additionally the cleaved crosslinker stub fragments (A/S/T) as stacked histograms.

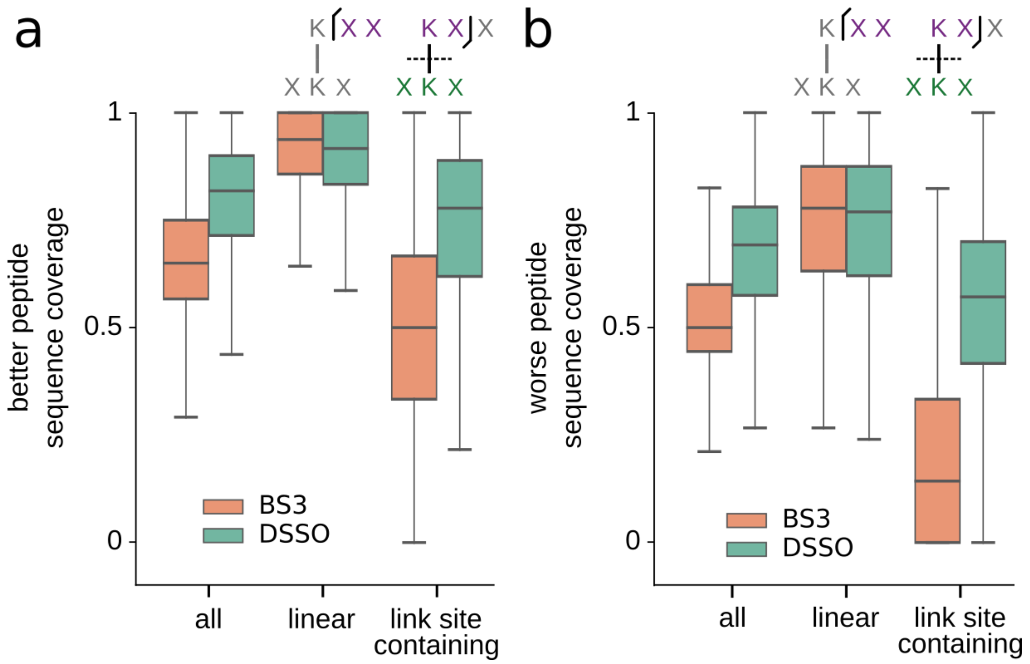

Figure S9. Sequence coverage of all, linear and link site-containing fragments (CSMs: n=1437) split into the better (a) and worse (b) fragmenting peptide. Boxplots depict the median (middle line), upper and lower quartiles (boxes), and 1.5 times the interquartile range (whiskers).

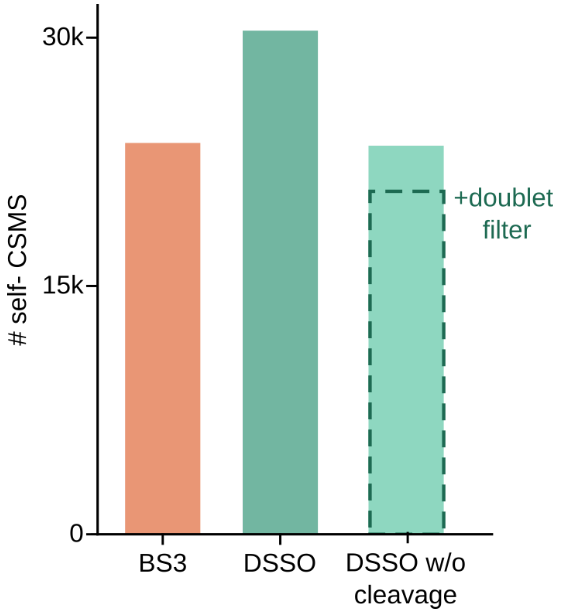

Figure S10. Number of self-CSMs passing 5% CSM-level FDR for BS3 and DSSO. DSSO was additionally searched as a non-cleavable crosslinker and filtered for the presence of peptide doublets.

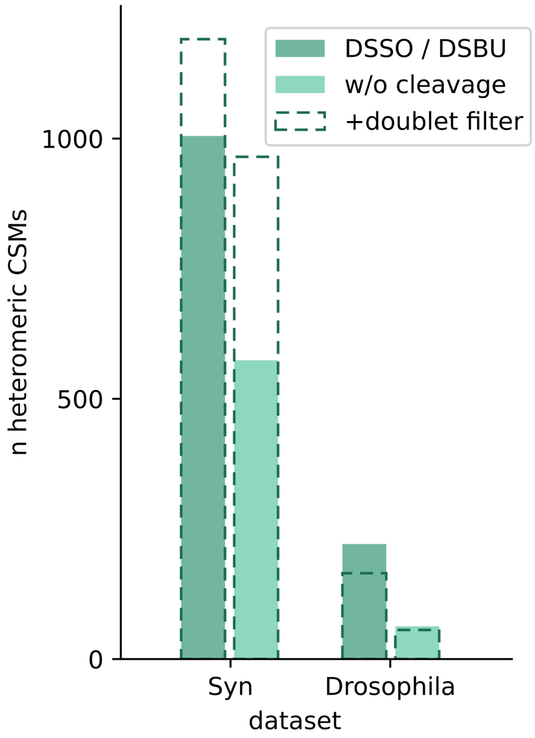

Figure S11. Number of heteromeric CSMs passing 5% CSM-level FDR for the Synapse and Drosophila datasets. DSSO was additionally searched as a non-cleavable crosslinker and filtered for the presence of peptide doublets.
